# Animals to heal animals: ethnoveterinary practices in semiarid region, Northeastern Brazil

**DOI:** 10.1186/1746-4269-5-37

**Published:** 2009-11-26

**Authors:** Maine VA Confessor, Lívia ET Mendonça, José S Mourão, Rômulo RN Alves

**Affiliations:** 1 Departamento de Biologia, Universidade Estadual da Paraíba, Av. das Baraúnas, 351/Campus Universitário, Bodocongó, 58109-753, Campina Grande-PB, Brazil

## Abstract

**Background:**

Animal-based remedies constitute an integral part of Traditional Medicine and this is true in Brazil as well both in rural and urban areas of the country. Due to its long history, zootherapy has in fact become an integral part of folk medicine in the country. The use of these natural resources for medical purposes, however, is not restricted to human diseases treatment, being also widely used for the treatment of animal illnesses. Ethnoveterinary is a science that involves the popular practical knowledge used to treat and prevent animal diseases. This study documents ethnoveterinary practices in one local semi-arid region in Northeast Brazil and discusses the findings in the surveyed area.

**Methods:**

Information was obtained through the use of semi-structured questionnaires. A total of 20 respondents (09 men and 11 women) provided information on animal species and body parts used as medicine, information concerning the illnesses to which the remedies were prescribed were also obtained.

**Results and conclusion:**

Eleven animal species were used in the treatment of 11 diseases in the surveyed area. The species inventoried comprise 3 taxonomic categories: mammals (05), reptiles (04) and birds (02). The obtained results proves that the use of animals or their derived products as therapeutic resources to the treatment of animal diseases represent a common practice and is culturally important in the studied area. It is evident that the popular knowledge about the ethnoveterinary practices is, frequently, passed through generations. We also noticed that, besides the cultural aspects, the socio-economic context permeates the use of zootherapics, since these practices constitute an alternative to the medicines acquired in veterinarian pharmacies, which have a high cost.

## Background

Naturally derived substances of plant, animal and mineral origins have provided a continuing source of medicines since the earliest times known to man [1,2], and their use have been perpetuated through the use of traditional medicines. Traditional human populations have a broad natural pharmacopoeia consisting of wild plant and animal species [3,4].

Medicinal plants and animals have been used in virtually all cultures as a source of medicine [1-10]. The use of biological resources for medicinal purposes, however, is not restricted to human diseases treatment, being also widely used for the treatment of livestock diseases [11-20]. These uses are the study objectives of Ethnoveterinary medicine (EVM). This term was firstly used by McCorkle in the mid 1980's to designate the "people's knowledge, abilities, methods, practices and beliefs concerning animal health care" [21]. In contemporary society ethnoveterinary practices persist over all in rural areas, where the veterinary services are inexistent or inaccessible for the local population [14,15,21].

Although a number of ethnobiological inventories concerning the use of medicinal plants and animals in human health have been realized, the EVM is poorly described. This scarce description of the EVM resources are in stark contrast to the problems of livestock rearing, where the lacking regular access to essential medicines greatly hampers productivity. According to the FAO, the lack of drugs to treat diseases and infections causes losses of 30 to 35% in the breeding sector of many developing countries, where poor animal health remains the major constraint to breeding [22].

Plants comprise the largest component of the diverse therapeutic elements of traditional livestock health care practices [11-17,19-21]. Farmers and pastoralists in several countries use medicinal plants in the maintenance and conservation of the livestock healthcare. It is estimated that medicinal plants, for several centuries, have been widely used as a primary source of prevention and control of livestock diseases [23]. Besides plants, animal-derived remedies represent important resources used in EVM, however this use have been neglected in comparison to medicinal plant research.

The advent of modern medicine has in a certain way caused the decay of the use of EVM methods use [23], and traditional medicines have been substituted by "pharmacy remedies". However, the costs, the inaccessibility and other factors associated with the veterinarian system have contributed to the persistence of EVM. Nevertheless, associated with environmental degradation, traditional knowledge and culture including the practices aimed at the cure of animals have been lost through the decades [24]. Therefore, there is an urgent necessity to document traditional EVM knowledge, focusing on the maintenance of this important cultural practice.

Brazil is an outstanding country both because of its great wealth of genetic resources and complex cultural diversity [25]. The adaptation of the various human groups to the rich biological resources has generated invaluable local knowledge systems that include extensive information on plant and animal uses in general and medicinally useful species in particular [26-38]. Regrettably, studies about EVM knowledge are practically inexistent, even though this practice is widely spread in the country. Therefore researches relying this subject are essential to evaluate the importance of this theme, as well as to characterize the social-cultural context associated to this source of the biodiversity social use in the country.

The *Caatinga *(semi-arid vegetation) is a unique Brazilian biome with a considerable but poorly studied biodiversity closely associated with a diverse human cultural heritage [32-34]. In semi-arid region, animals and plants are widely used in traditional medicine and play a significant role in healing practices there. Although plants and plant-derived materials make up the majority ingredients used, whole animals, animal parts, and animal-derived products also constitute important elements of the popular medicine in the region [32-36]. In the semi-arid region of Brazil, Zootherapy (treatment of ailments with remedies made from animals and their products) form an integral part of the local culture, and information about animals and their uses are passed from generation to generation through oral folk lore [32]. In many areas (such as semi-arid region) this folk knowledge is rapidly disappearing - with traditional medicine being set aside in favor of modern medical practices, and veterinary drugstores are now frequently found in formerly isolated areas [18].

Zootherapeutical practices are used both for human and animals in the semi-arid region. Nevertheless, works concerning the theme are still scarce, and specifically about the animals used in EVM only one work was published [18]. The present study documents zootherapeutic practices of EVM in rural areas of the municipality of Pocinhos, located in the semi-arid region in the Northeast of Brazil. Documentation on zootherapeutical practices can assist in protecting traditional knowledge, and in ensuring that future users recognize the contributions made by traditional communities, the current custodians of traditional knowledge.

## Materials and methods

### Study area

**Figure 1:**
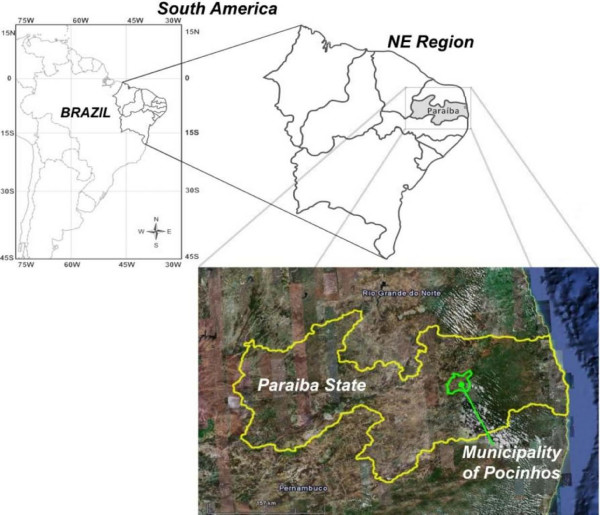
**Map of study area, Municipality of Pocinhos, NE, Brazil**.

The present study was carried out in the municipality of Pocinhos (7°04'36"S × 36°03'40"W), located in the western sector of the Borborema Plateau, Paraíba State, Pocinhos micro-region, Paraibano meso-region, Brazil [23] (Figure 1). The municipality of Pocinhos is bordered by Campina Grande, Boa Vista, Puxinanã, Soledade, Olivedos, Barra de Santa Rosa, Algodão de Jandaíra, Esperança, Areial, and Montadas [39]. The municipal seat is situated at an altitude of approximately 646 meters above sea level, and is located 132 Km from João Pessoa with access through the BR 230/PB and 121 highways [40].

Pocinhos occupies an area of 630 km^2 ^and has a population of 14 880, of which about fifty percent (7,323) reside in the rural zone [41]. The average annual temperature is 23°C and varies little during the year. The region has a very low rainfall rate, oscillating annually between 400 and 600 mm. The climate is hot, semiarid, with rainfall in the autumn and winter months (dry Mediterranean type) [39] and the vegetation is dominated by sub-deciduous and deciduous forests typical of semi-arid regions. Soil quality is very variable, with a certain predominance of average to high fertility areas [39]. The regional topography presents a rolling landscape with deep, dissected, and narrow valleys.

Subsistence agriculture and livestock husbandry constitute the main economical activities in the municipal district. It is also common the use of animals (including wild animals) as pets [33]. The community of Pocinhos city is formed by typical Sertanejos peoples, descending from settlers families of the Agreste mesoregion of Paraíba.

### Procedures

The field study was carried out from July 2007 to December 2008, and information on the use of medicinal animals used in EVM was obtained through semi-structured questionnaires, complemented by free interviews and informal conversations [42]. The interviews were individually carried out and, during the first contacts with the local population, "native specialists" were identified, in other words, people who consider themselves, and are considered by the community as having exceptional knowledge about the use of animals to ethnoveterinary purposes. Twenty people (09 men and 11 women) were interviewed. Among these interviewees, 10% were aged 21-40 years old, 40% were 61 years old or more and half of the sample (50%) were in the 41-60 age range. The knowledge about medicinal products obtained from animal is widespread in region, primarily amongst the elderly; they are natural retainers of traditional knowledge in their respective communities.

To determine the relative importance of each local known species, their use-values were calculated (adapted from the proposal of Phillips et al. [43] using the following formula: UV = ΣU/n, where: **UV **is the use-value of a species; **U **the number of citations of that species; and **n **the number of informants. The use-value of each species is based only on the importance attributed by each informant and does not depend on the opinion of the researcher or the interviewees.

## Results and Discussion

The results reveal that EVM is an activity mainly orally passed through generations, specially from father to child and constitutes part of the culture of the people who live in the *Caatinga *region, as noticed through passages of some interviewees' testimonies: ".we learn from the old people, it passes from father to child." male, 76; ".it's a parents' tradition heritage." male, 72. In some cases, however, the obtained ethnoveterinary knowledge is derived from friends, neighbors, zootechnists from the region and also from TV programs.

**Figure 2:**
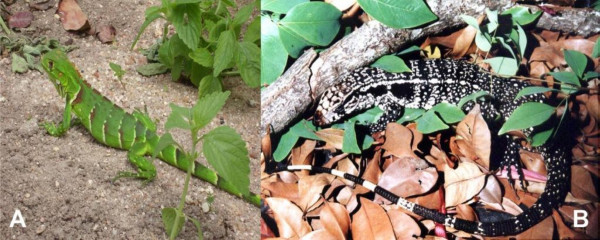
**Examples of animals used in Ethnoveterinary medicine, semi-arid region, NE Brazil, A - *Iguana iguana *(Common Green Iguana)(Photo: Maine V. A. Confessor); B - *Tupinambis merianae *(Lizard, "teju") (Photo: Yuri C.C. Lima)**.

**Figure 3:**
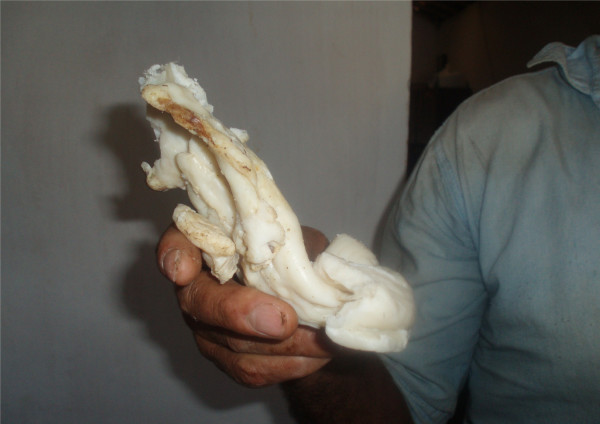
**Suet derived of the *Ovis aries *(Ram) used in Ethnoveterinary medicine in municipality of Pocinhos, semi-arid region, NE Brazil**.

Eleven animal species were used for ethnoveterinary purposes in the surveyed area. The inventoried species comprise 3 taxonomic categories: mammals (05), reptiles (04) and birds (02). The most important medicinal species were: "Teju" lizards (*Tupinambis merianae *(UV = 0.55); iguana (*Iguana iguana*) (UV = 0.45); pig (*Sus scrofa Domesticus) *(UV = 0.4), and rattlesnake (*Crotalus durissus *Linnaeus, 1758) (UV = 0.35). The medicinally used animal parts were fat, feathers, horns, eggs, bones, milk and leather (Table 1). Examples of animal species and zootherapeutic product used as medicine are showed in Figure 2 and 3. All animals recorded in the present study are also utilized in EVM in the municipality of Cubati, also localized in the semi-arid region [18] and in some cases are used to treat the same illnesses, for example, the fat of the fox (*Cerdocyon thous*) is used by the local residents for the treatment of the uterus prolapse, "mother's body" as they used to call.

**Table 1:** Zootherapeutics resources used in Ethnoveterinary medicine in municipality of Pocinhos, semi-arid region, NE Brazil.

Famíly/species/local *name*	UV	Part used	Disease (or illness)
Suidae *Sus scrofa domesticus (*Linnaeus, 1758) - Pig "Porco"	0.4	Fat	Scabies; Skin diseases; swellings
Phasianidae *Gallus gallus domesticus *(Linnaeus, 1758) - Domestic chicken, "Galinha"	0.15	Fat Eggs	Inflammation; "Estrepes" (suck a splinter out of skin); poisoness
Tinamidae *Nothura maculosa cearensis *Naumburg, 1932 - Spotted Nothura, "Codorniz"	0.25	feather	Snakes bite
Teiidae *Tupinambis merianae *(Duméril & Bibron, 1839) - Lizard teju, tejuaçú"	0.55	Fat	Thorns; Pain; Furunculosis; Inflammation "Estrepes" (suck a splinter out of skin); Eyes problems; Swellings
Iguanidae *Iguana iguana *(Linnaeus, 1758) - Common Green Iguana, "Camaleão"	0.35	Bone Fat Leather	Thorns; Furunculosis; Swellings; "Estrepes" (suck a splinter out of skin)
Dasypodidae *Euphractus sexcintus *(Linnaeus, 1758)--armadillo, "tatu peba"	0.05	Fat	Furunculosis
Viperidae *Crotalus durissus *Linnaeus, 1758 - South American rattlesnake, "Cascavel"	0.35	Fat	Tumor; Wounds; Snakes bite; Skin problems
Canidae *Cerdocyon thous *(Linnaeus, 1766)-- fox, "raposa"	0.25	Fat	Tumor; Uterine prolapse; Wounds; Skin problems
Bovidae *Bos Taurus *Linnaeus, 1758 - Domestic cattle, "Gado/boi/vaca"	0.15	Horn Milk	Snakes bite; Poisoness
*Ovis aries *Linnaeus, 1758 - Ram, "Carneiro"	0.15	Fat	Burns; Bone fracture; Thorns
Chelidae *Phrynops geoffroanus *(Schweigger, 1812) - Geoffroy's side-necked turtle, "cágado d'água"	0.1	Fat	Swelling; Tumor

**Figure 4:**
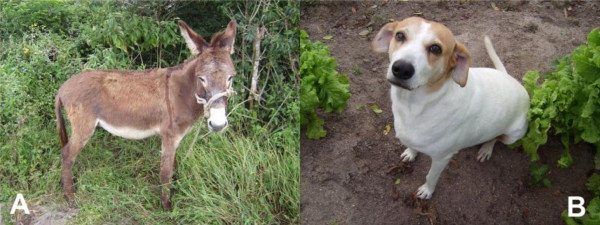
**Examples of animals treated in Ethnoveterinary medicine, semi-arid region, NE Brazil, A - *Equus asinus *(asino); B - *Canis lupus familiaris *(dog) (Photos: Maine V. A. Confessor)**.

These species were used to treat 11 different diseases. The most cited diseases were: tumors, furuncunlosis (n = 9 citations); snakes bites (n = 8); thorns (n = 5); wounds (n = 4); scabies (n = 4); "Estrepes" (suck a splinter out of skin), (n = 4); and harmed areas (n = 3). The diseases quoted during the interviews affect as much as livestock animals and pets, some of them are: cow/cattle, sheep, goat, horses, dogs, pigs. Examples can be viewed in figure 4. Most of the interviewees affirm that the zootherapics can be used to cure diseases that affect all domestic animals and pets.

Zootherapeutics are usually applied in simple ways, mostly through ingestion or direct application to the affected area and usually not in association with other animal derived ingredients. In some cases, however, an association with medicinal plants or other resources is observed as, for example, for the treatment of snake bites using the "Cordoniz" (*Nothura maculosa) *feather in association with garlic (*Allium sativum*), or the ox (*Bos Taurus) *horns with garlic and cashew nut of "Cajueiro" (*Anacardium occidentale)*. Other example is the treatment of scabies using the pig fat with sulfur generally acquired in market or pharmacy.

It is known that the use of medicinal animals is frequent in several countries in Latin America [9,44-49], and that often there are overlaps in the medicinal use of plants and animals in traditional medicine for humans [9,44]. Similarly, in EVM, this overlaps also occurs. In semi-arid areas of Brazil, medicinal plants are used in association with animals for ethnoveterinary utilization. This is not surprising given the use of animals products is common in EVM, although works relying the theme are still scarce. Barboza et al. [18] recorded the utilization of animals (zootherapeutics) as sources of medicines in folk veterinary medicine (ethnoveterinary) in the semi-arid northeast region and verified that 15 animals are used in the prevention or cure of animals' illnesses in that region. So that, it is assumed a close association between phytotherapeutic and zootherapeutic practices in traditional medicine, such as for human's usage as animals'.

Animal and human medicine has been closely linked throughout history. Some diseases affect animals and people and can be treated with similar remedies. This way, many plants used in EVM are also used for treatment of human diseases. In Brazil, for instance, São Caetano's melon - *Mormodica charantia *L., the purga potato - *Operculina hamiltonii *and the Pumpkin's seed - *Cucurbita pepo *L. are used as vermifuge in veterinary medicine, probably similarly the same way they are prepared in human medicine [50]. A similar trend has been observed in the present study for the use of medicinal animals, once that in several instances the treatment of animal illnesses was based on the healing of similar human diseases, as confirmed by the interviewees and also as reported in the literature [18]. For instance, the fat of *Ovis aries *is used for the treatment of bone fracture and thorns in veterinary medicine, similarly the same way they are prepared in human medicine [9]. These results support other studies which have revealed that in most traditional societies, there is no clear division between veterinary and human medicine [51].

Scarpa [52] reported a strong parallel among the plants used in the traditional veterinary medicine of the chaco in Argentinean northwest, where 60% of the reports stated as therapeutic, showing an identical correlation in human medicinal therapy. Such similarities of uses by several plants in EVM and in human ethnomedicine links the fact that ethnoveterinary practices have probably followed two main evolutionary pathways: one based on the observations of self-medication in animals, and the other related to *human folk medicine*, as reported by Pieroni et al. [53]. The use of folk remedies in animals to treat diseases or conditions based on similar or identical illness that attack humans is named by Barboza et al. [18] of "human model for diseases in animals". The relationships between etnoveterinary and human ethnomedicine on this perspective can be easily explainable, since mammals are the main stock animals (e.g. cattle, sheep, goats, pigs, among others). Regarding this, animals usually face health problems that also affect humans, being noticed in identical manners by several people or communities. However, this cannot be taken as rule for all particular cases.

The catalogued animals in the present study are common in the surveyed area, this way it is evidenced that the fauna composition of *Caatinga *influences the choice of medicinal animals' usage. For example rattlesnake, iguanas, and the "*Teju" *lizards are reptiles frequently encountered in the local *Caatinga *fauna. Some domestic animal species that are commonly raised in region are also used to produce materials for traditional medicine, e.g, pigs, cows (*Bos taurus*). A similar tendency was reported by Adeola [54], who observed that the animals used for preventive and healing medicine were associated with the natural area in which the users live, as well as with their relative species abundance. The use of accessible local resources is closely linked to historical factors and medicinal knowledge traditionally focuses on local species, reflecting the transmission of knowledge through many generations, while financial restrictions limit access to exotic resources [27,55,56].

In surveyed area, the local medicinal fauna is largely based on wild animals. A similar situation was described by Adolph et al. [57] in Sudan, where wild animals such as porcupines, ostriches, sitatunga, hyena, termites and vultures play their part in disease treatment. Vultures are the main ingredient used to cook a soup which is given to sick cattle. In the same way that chicken soup has been found to have a clumping effect on leukocytes and thus act as a common cold remedy, so perhaps vulture soup does the same.

Our results demonstrated the persistence of folk veterinary medicine practices in the surveyed area, that are being influenced by culture and social-economic aspects, constituting an alternative to the remedies acquired in veterinarian pharmacies that have a high cost. Other studies are also necessary to preserve the popular medicinal knowledge which is important to enhance our understanding on the relationship between human, society and nature, and also to elaborate more effective strategies for conserving natural resources especially to the Caatinga biome, where the studies concerning this subject are scarce. As pointed by Mathias and Perezygrovas [55], promoting ethnoveterinary medicine means helping local people use their experience and knowledge to build on their own strategies and resources.

The medicinal use of animal-derived remedies for treating various disorders in humans and in their animals is a centuries-old tradition in many cultures [3,4,9]. The possible benefit of animal-derived medications constitutes a rewarding area of research, particularly in countries such as Brazil which have a rich biodiversity of animals resources coupled with a high prevalence and variety of infectious diseases.

Ethnoveterinary medicine may play a significant role in the management of such diseases in a cost-effective and accessible manner, and there are an increasing number of publications in the scientific literature reporting on the ethnopharmacological activity of plants used in traditional veterinary medicine. Unfortunately, little research has been done so far to prove the claimed clinical efficacy of animal products for medicinal purposes [58,59]. Nevertheless, the chemical constituents and pharmacological actions of certain animal products are known to some extent, but more ethnopharmacological studies focusing on animal remedies are needed in order to better define the eventual therapeutic usefulness of this class of biological remedies as well as the toxicity.

The protection and revival of traditional medicine knowledge and practice in thousands of ethnic communities is an important mean of providing affordable and sustainable healthcare. Traditional health practitioners and farmers' knowledge can be useful to identify, implement and manage medicinal resources conservation.

The literature on EVM is incipient in Brazil, evidencing the necessity of new studies, principally considering the cultural, socioeconomic and ecological importance associated to biological resources' usage in Ethnoveterinary medicine, seeking to the cultural maintenance and the sustainable use of fauna and flora involved in that use manner. Conservation of both biological and cultural diversity in Brazil can also value and preserve the different human societies that exist in this region. The need to conserve and protect the world's medicinal animals and plants is required not only for man but also for his domesticated animals.

## Competing interests

The authors declare that they have no competing interests.

## Authors' contributions

MVAC, LETM, JSM, and RRNA - Writing of the manuscript, literature survey and interpretation MVAC and LETM - Ethnozoological data, literature survey and interpretation; RRNA - Analysis of taxonomic aspects. All authors read and approved the final manuscript.
